# The Anatomy of the Cranium and Face

**Published:** 1868-03

**Authors:** Claude Baxley

**Affiliations:** Professor of Materia Medica in the Maryland College of Pharmacy, and Demonstrator of Anatomy in the Baltimore College of Dental Surgery.


### AMERICAN JOURNAL OF DENTAL SCIENCE.

Vol. I. THIRD SERIES--MARCH, 1868. No. 11.

### ORIGINAL COMMUNICATIONS.

### ARTICLE I.

### *The Anatomy of the Cranium and Face.*

By Claude Baxley, M.D.,

Professor of Materia Medica in the Maryland College of Pharmacy, and Demonstrator of Anatomy in the Baltimore College of Dental Surgery.

There is perhaps no portion of human anatomy of greater interest, and especially to the dental student, than that which relates to the *cranium* and *face*. The first as the protecting envelope of that wondrous organ of intellect, about whose delicate convolutions there yet remains so much of mystery; and the second, as comprising in its osteological architecture the orifices for the lodgement of the organs of the senses, which, surrounded by a movable mask of muscles, possessed of varied offices, constitute by their configurations and proportions, what we ordinarily denominate the *features*.

The bony and muscular developements of the face, together with the skin and fatty tissue, comprise in their variations, some of the striking characters by which we learn to recognize the sex, the age, and the different races of mankind, as well as in individuals, the moral, physical and intellectual traits.

For example—the marked delicacy of feature, and greater abundance of cellular and adipose tissues, indicate at a glance the *female* face. Again, the well known peculiarities in the distribution of the bones, particularly in the absence of teeth, the redundance of fat, and the slight muscular developement, in the plump and inexpressive face of *infancy*—whilst in the *aged* we have the reverse of this, the furrowed features and the pinched expression occasioned by the deficiency of fat, and the prominence of firm and rigid muscles.

And as time writes unerringly his wrinkle on the brow, so, no less surely do the traits of character stamp their impress on the face, which indeed might well be termed the "mirror of the soul."

Most of the superficial muscles of the head and face, differ in several particulars from those of other regions; they are destitute of that dense fascia which constitutes so uniform a covering to other muscles; and with a few exceptions, there is no tendinous structure entering into their formation. They lie closely attached to the skin, and their fibres are delicate and pale, so that their dissection is often tedious, and to the inexperienced, one of considerable difficulty. Regarding their function, these muscles are in part both voluntary and involuntary, and therefore come under the class of such as are termed mixed muscles.

Opposite the orbitary and buccal cavities of the skull, the skin presents transverse eliptical openings, which under the influence of will, may be tightly closed, or widely opened; each of these cavities is surrounded by a particular orbicular muscle, which, by its contraction, closes the orifice that it encircles; but here its capacity ends, and the office of opening or dilating the orifice belongs to other muscles, which radiate as it were from these sphincters, their outer extremities being inserted into adjacent osseous surfaces, from which positions they are enabled to perform the double motions which dilate the orifices, and expand the features generally.

The first of the orbicular muscles to be considered is the *orbicularis palpebrarum*, or *orbicularis oculi* as it is sometimes termed. This is a thin membranous muscle, situated immediately beneath the skin, in front of the orbit, and around the edge of which it extends considerably in all directions, except inwards, it is therefore somewhat oval in shape, and is depressed in the centre, that it may conform in its configuration to the globe of the eye. It has dividing it, the horizontal cleft we have above referred to, and is formed of concentric circles around this opening. There are several origins to this muscle, the first, from the upper end of the nasal process of the superior maxillary bone, the next from the adjacent internal angular process of the frontal bone, and the last from the upper margin of the palpebral ligament. This ligament, called also the *tendo* oculi, is nearly a fourth of an inch long, attached internally to the upper border of the nasal process of the superior maxillary, and passing outwards and backwards, to the internal commissure of the lids, divides into two slips, between which lies the caruncula lachrymalis, and are inserted each into the tarsal cartilage, and the lachrymal duct. From these three origins that we have given, the orbicularis palpebrarum forming a broad fasciculus of fibres, passes over the superior tarsal cartilage and outwards, to the external angle of the eye, then curving inwards, cover the lower tarsus, terminating at the inner canthus, where it is inserted into the margin of the orbital process of the superior maxillary bone, also its nasal process, and the inferior margin of the palpebral ligament. The external fibres of this muscle are strong and red, while those that are within, lying upon the eye lids, are thin, scattered and pale.

Regarded physiologically, the orbicularis palpebrarum differs from all the voluntary muscles; its mechanism is very plain, and its office is simply to protect the eye, this it accomplishes in several ways. A slight contraction of the muscle closes the eyelids, a more powerful contraction draws along with it the adjacent radiating muscles, causing the depression of the brows, and the ascent of the cheeks, giving the whole face a puckered appearance; it is the external, stronger and more voluntary fibres that act in this way and it is they that are chiefly used in knitting the brows, and drawing them down to shade the eyes. The voluntary and involuntary character of this muscle, is founded no less in anatomy than in physiology, so that it might indeed be considered as two muscles.

The simple occlusion of the eyelids, which we call *winking* is performed by the contraction of the inner and more delicate palpebral portion of the muscle, especially the superior. Regarding its nervous excitability, and the operation of its functions in the opposite conditions of *sleeping* and *waking*, the workings of this muscle are of particular interest. Whilst awake, the ciliary portion of the orbicular muscle is ordinarily relaxed, still it is under the command of the will, though it remains to a remarkable degree under the influence of the organic instinct that prompts to self preservation ; for instance, at intervals the eye lids rapidly close, and are as instantaneously reopened, for the purpose of lubricating the ball of the eye ; and whenever that delicate organ is threatened with the contact of a foreign body, it contracts in advance of, or in spite of the will. When sleep approaches, the contraction of this muscle closes the eye-lids, and so long as sleep continues, it remains contracted, whilst all other muscles of the body, are in a state of relaxation, and it only relaxes again at the moment of awaking. Another interesting fact regarding the function of the muscle, is, that if a bright light suddenly falls upon the countenance of the sleeper, the outer fibres will also be instantaneously contracted, so as to give additional shading and protection to the eye.

In sleep the eye is covered principally by the descent of the upper palpebra, hence the "equator oculi" in real sleep, is below the median line, such is not the case when sleep is feigned.

Another muscle, lying immediately beneath this last mentioned, though quite small requires a passing remark, as a participant in the functions of the orbicularis; it is the *corrugator supercilii*—a short though somewhat thick muscle, arising from the inner part of the nasal protuberance, its fibres, of a conical form, run upwards and outwards, and blend with the deeper portions of the orbicularis palpebrarum opposite the middle of the brow, which it draws downwards and upwards, knitting it as it were, and wrinkling the forehead vertically, thus contributing to the expression of ill-temper or discontent.

The second sphincter muscle, the *orbicularis oris* is of no less interest than the first; situated between the skin and mucuous membrane, it is of considerable thickness, and encircling the mouth, is of transversely oval form, composed of two semi-elliptical halves, separated by the cleft of the mouth, beyond the commissures of which the fibres from the upper and lower portions blend, and encroach upon the cheeks. Unlike any of the other face muscles, the orbicularis oris has no bony connections, and is literally suspended amid the muscles that radiate towards it. When the mouth is closed, the fibres of this muscle mutually converge, and are therefore puckered in the line of their direction; their relaxation passively permits the expansion of the lips. The fibres directly adjacent to the edge of the mouth, form a particular investing fasciculus, which is turned back, or folded outwards, and it is the development of these fibres which produces that marked eversion of the lips characteristic of the negro race. Like the sphincters generally, the chief action of this muscle is to contract from its circumferance towards its centre, but it is distinguished from these by the very remarkable power, peculiar to it alone, of contracting in isolate portions, one lip, or one angle of the muscle may act independently, and even one side of the lip may move, the rest remaining quiescent; thus aided by other muscles, this capacity enables it to participate in the play of numberless expressions of the face.

In reviewing those muscles which surround the orbicular muscles that we have just considered, we have first to notice the *occipito frontalis*, a thin, though broad and rather extensive muscle digastric or rather quadriceps, situated on the upper part of the cranium, which it closely envelops from the two superior curved lines of the occipital bone, to the supra-orbital arches of the frontal bone. It is composed of two distinct muscular bellies, united by an epicranial aponeurosis, and hence by some anatomists has been regarded as two different muscles. It is with the anterior or frontal portion that we have most to do, as connected with the obicularis palpebrarum. It is irregularly quadrilateral in form, and its fleshy fibres proceeding from the epicranial aponeurosis over the coronal suture, descend parallel with one another, towards the eye-brows, where they intermingle with those of the orbicularis; a few fibres of the inner portion being continuous over the nasal bones, constitute a little muscle called the *pyramidalis nasi*. The occipito frontalis is very closely adherent to the scalp, and but loosely attached to the cranium, over which it moves easily and to a considerable extent, thus forming a wise provision of nature for the protection of the integument, which with the scalp, will yield gradually before any weight or pressure against the head, rather than be lacerated by it.

Its office is to elevate the integuments of the forehead into transverse wrinkles, raise the eye-brows and cause them slightly to diverge, making tense the skin of the upper lids, and thus exposing the eye-ball as in the act of staring. If the anterior portion be fixed, then the action of the muscle is to draw the scalp downwards and forwards.

The use of the *pyramidalis nasi* is much the same, it is inserted into the compressor nasi muscle as it spreads over the nose, and by its contraction elevates the integuments of this region of the face, though to a very slight degree, its chief office being, to serve as a fixed point to the frontalis.

The muscles next concerned to a considerable extent in the play of expression, are those thin delicate fleshy ribbons situated between the orbicular muscles of the eye and mouth; and first, the *levator labii superioris alæque nasi*, whose name much larger than itself would prove not a little inconvenient, did it not so clearly indicate its location and its use. Then, as we have said shaped like an acute angled triangle, this muscle is placed obliquely on either side of the nose, arising by a tapering summit from the nasal process of the superior maxillary bone, it descends obliquely outwards, and becoming broader, its inner edge is attached to the cartilage of the ala nasi, and also to the integument; whilst the outer portion expands into superficial fibres, which are attached to the skin of the upper lip, and into deeper fibres, which blend with those of the levator labii proprius and orbicularis oris. By its name and attachments, the action of this muscle is easily understood, as an elevator of the alæ of the nose and the upper lip, giving to the face that characteristic expression which has entitled it the *muscle* of *disdain*.

Immediately outside and parallel with the last mentioned, is the *levator proprius labii superioris;* this is irregularly rectangular, and like its neighbor is thin and ribbon like, extending over the middle part of the face, it is attached above by a few small aponeurotic fasciculi to that portion of the superior maxillary bone, and of the malar bone which forms the inferior edge of the base of the orbit, from this origin, its fibres converging slightly as they descend obliquely inwards, into the upper segment of the orbicularis oris, which it assists in elevating, at the same time drawing it slightly outwards.

Still external to the levator proprius, are two similar muscles passing diagonally over the cheek from without inwards, the first of these, the zygomaticus minor, arises from the upper part of the malar bone, and from within the circumferance of the orbicularis palpebrarum, and below, loses itself in the skin and the orbicularis oris, thus we see that the thread like fibres of the zygomaticus minor directly connect the two orbicular muscles, aud excites them to simultaneous contraction; occasionally, this little muscle is wanting. Its fellow, however, the *zygomaticus major* is always present; and is considerably larger, being situated on the outside and a little under the minor, it runs in the same direction, to become blended with the outside of the commissure of the orbicularis oris, and also draws that muscle upwards and outwards.

The *caninus* or *levator anguli oris* is the last muscle that we shall notice as connected with the upper portion of the orbicular muscle of the mouth. Its name designates its origin in the middle of the canine fossa, and also indicates its office for it is inserted at the outer portion of the labial commissure, and besides elevating the angle of the mouth, also draws it slightly inwards, thus contributing to important modifications of feature.

We will now glance for a moment at the antagonists of these levator muscles; we find them in the *depressors* of the lip; and first at the *quadratus menti, or depressor labii inferioris,* this muscle is of rhomboidal form, its pale fibres proceed inferiorly from the inner half of the external oblique line of the inferior maxillary, and from the platysma myoid, of which indeed it is a continuation, for though there is an aponeurotic intersection which indicates the line of separation at the base of the jaw, between the two muscles, yet even this is traversed by a few muscular fibres, which establish the continuity of these muscles; the fibres running upwards, also converge, the deeper portions losing themselves in the deep part of the semi orbicular, the more superficial, passing in front of this muscle, are attached to the integument of the lower lip; the inner fibres at their upper part decussate with those of the opposite side; and this interlacing of its fibres enables it not only to draw its own side obliquely outwards, but also to pull upon the other half, so as to bring the whole of the lip towards itself. In the simultaneous action of the two quadrati, the lower lip is stretched transversely, rather than depressed.

The last muscle that we shall consider, the *triangularis*, or *depressor anguli oris*, is situated subcutaneously at the outer side of the quadratus menti, it is a thin muscle and as its name indicates of triangular shape, directed vertically, and arises by a broad base, from the edge of the lower jaw, its converging fibres ascend towards the commissure of the lips. It is closely connected with the skin, and a few of its deep fibres mingle with the orbciularis, and superiorly even blends its fibres with the levator anguli oris, and zygomaticus major. In its action, it is therefore the antagonist of these muscles ; they are also mutually opposed in the part they perform in the play of the features ; for the triangularis gives expression to the sorrowful emotions, as the others are the agents of livelier feelings.

It would be impracticable in an article such as this, to refer to all the muscles of the face, which to a greater or less degree play their part in the anatomy of expression, we must therefore rest satisfied with this casual survey of those most prominent. As portrayers of the passions and emotions of the human heart, and as faithful indexes of the various traits of character, they are full of interest, for truly was it said that "the soul fashioneth for herself a habitation fit to dwell in."

